# Bilateral Postaxial Polydactyly of the Feet in an Adult: Surgical Management and Outcomes

**DOI:** 10.7759/cureus.101428

**Published:** 2026-01-13

**Authors:** Ammar Aljefri, Omar A Batouk, Ahmad A Ismail, Zameer Ahmad, Abdulaziz Saber

**Affiliations:** 1 Orthopedic Surgery, King Abdulaziz Medical City, Jeddah, SAU; 2 Orthopedic Surgery, Ministry of National Guard – Health Affairs, Jeddah, SAU; 3 Orthopedics, King Abdulaziz Medical City, Jeddah, SAU

**Keywords:** bilateral, case report, foot deformity, polydactyly, postaxial, surgical excision

## Abstract

Bilateral postaxial polydactyly of the feet is a rare congenital anomaly characterized by the presence of extra digits on the lateral aspect of both feet. While polydactyly is a relatively common limb malformation, isolated bilateral involvement of the feet, particularly type A variants with bony elements, is uncommon. We report the case of a 33-year-old male with bilateral type A postaxial polydactyly who presented with pain and shoe-wear difficulties. Radiographs confirmed the presence of small osseous fragments within the accessory digits. The patient underwent staged surgical excision, first on the right foot and subsequently on the left, using a racquet-shaped incision for complete removal of the digit and associated soft tissue. Both procedures were uneventful, and the patient achieved excellent functional recovery and cosmetically satisfactory outcomes with no postoperative complications. This case highlights the importance of individualized management of postaxial polydactyly based on morphology and symptoms. It also reinforces that, even in adulthood, surgical excision offers significant symptom relief and improvement in quality of life. Given the rarity of isolated bilateral cases, this report contributes to the limited body of literature on the surgical management and outcomes of this condition.

## Introduction

Bilateral postaxial polydactyly of the feet is a rare congenital condition characterized by the presence of extra digits on the lateral side of both feet [1]. This anomaly is classified as type B when the additional digit is underdeveloped and consists primarily of soft tissue, while type A involves a more functional, bony structure [1]. Polydactyly, in general, is a common limb abnormality, but bilateral involvement of the feet is much rarer, especially in isolated cases. The prevalence of polydactyly varies significantly among populations, being more common in African and African American groups compared to Caucasian or Asian populations [2]. Most cases follow an autosomal dominant inheritance pattern [2]. Polydactyly may occur in isolation or as part of syndromes such as Ellis-van Creveld syndrome, Bardet-Biedl syndrome, or Meckel-Gruber syndrome, where additional systemic anomalies such as cardiac, renal, or neurological defects may be present [3]. Embryologically, polydactyly arises from disruptions in the SHH signaling pathway, which regulates the anterior-posterior axis of limb development. Misregulation of this pathway can lead to the formation of extra digits in the postaxial (fibular) regions [3,4].

Previous studies have noted that while postaxial polydactyly is relatively common in the hands, bilateral involvement of the feet is quite rare, especially in the absence of syndromic features. A study conducted by Castilla et al. (1973), which reviewed congenital anomalies in South America, noted a much lower incidence of bilateral polydactyly of the feet compared to unilateral cases [5]. Another study conducted by Chiang et al. (1997) reviewed the cases in their clinic over a 15-year period and also found a much lower incidence of bilateral polydactyly compared to unilateral cases [6].

More recent literature supports these findings. Chocron et al. (2021) described lower extremity postaxial polydactyly in a surgical series and noted that true bilateral, non-syndromic foot involvement is uncommon [7]. Kelly et al. (2021) provided a comprehensive review of foot polydactyly, reporting that bilateral cases constitute only a small fraction of all reported cases [8]. From a surgical perspective, Lui (2013) recommended a racquet-shaped incision for metatarsal-level postaxial excisions to achieve favorable cosmetic and functional outcomes in older patients, which supports the approach applied in this report [9]. Cheng et al. (2021) described bilateral postaxial polydactyly associated with hallux valgus in an adult patient and demonstrated that tailored surgical planning provides excellent outcomes [10].

The justification for this study lies in the limited number of documented cases of bilateral postaxial polydactyly of the feet, especially in isolated, non-syndromic presentations [5,6]. Given the rarity of this condition, the study can contribute to expanding the understanding of its genetic and clinical features, thus aiding in diagnosis and management. Moreover, many reports focus on polydactyly of the hands or unilateral foot involvement, leaving a gap in the literature regarding the treatment outcomes and management strategies for bilateral cases. This study is significant as it provides insight into surgical management and potential long-term outcomes, which are critical for clinicians when encountering similar cases. Early diagnosis and proper management are essential for both cosmetic and functional reasons, especially in cases where surgical intervention is necessary to restore foot biomechanics and prevent complications in walking and weight-bearing activities [1,6].

## Case presentation

A 33-year-old male with no known medical illnesses or significant past surgical history presented to the orthopedic clinic in July 2024 with bilateral postaxial polydactyly. He had an additional sixth digit on the lateral aspect of both feet since birth. The patient reported increasing discomfort, particularly when wearing shoes, as well as occasional pain, exacerbated by prolonged standing or walking.

**Figure 1:**
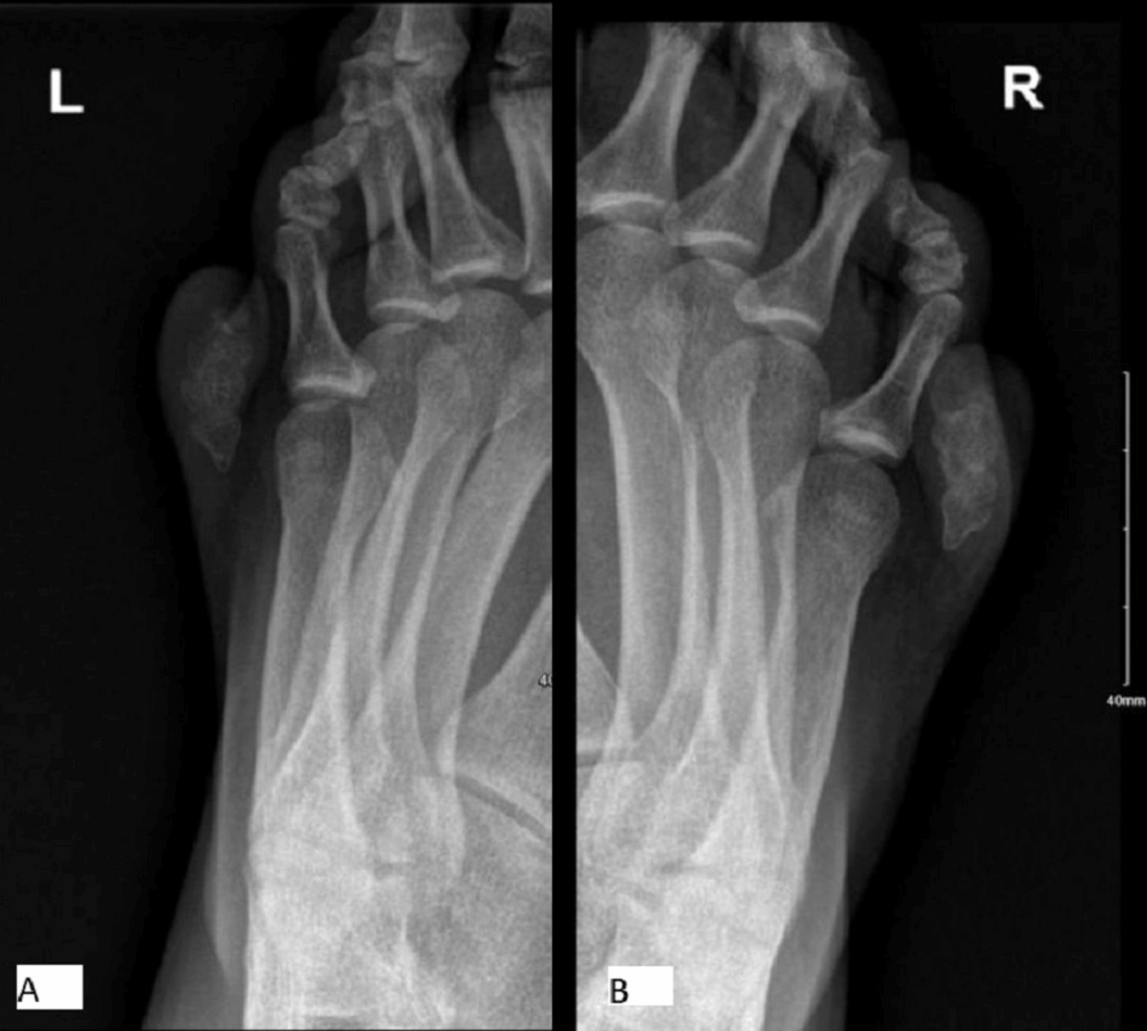
Bilateral foot X-rays showing ossification of the extra digits. (A) Left foot. (B) Right foot.

At the initial clinic visit, the patient underwent a full physical examination. Both extra digits were fully formed with limited movement, though they did not functionally contribute to gait or balance. Plain radiographs of both feet confirmed the presence of small osseous fragments associated with the extra digits. Blood work was also obtained at this time, and all results were within normal limits (Figure 1).

After discussing treatment options with the patient, surgical excision was recommended due to the discomfort and functional limitation caused by the extra digits. The patient was counseled regarding the risks and benefits of surgery, including the potential for infection, scarring, and neuroma formation. A staged approach was planned, starting with the right foot, followed by the left foot after a month, to allow for recovery and facilitate ambulation. The patient consented to the surgical plan and received preoperative and postoperative instructions, including wound care and pain management.

Surgical procedures

Right Foot Surgery (August 2024)

The patient was seen in the Pre-Admission Clinic (PAC) before surgery, where he was evaluated and cleared for the procedure. In August 2024, he underwent surgical excision of the postaxial extra digit on the right foot. The procedure was uneventful. After admission to the operating room, 2 g of prophylactic cefazolin was administered, and general anesthesia was induced. A tourniquet was applied to the proximal thigh, and the foot and ankle were prepared in a sterile fashion.

**Figure 2:**
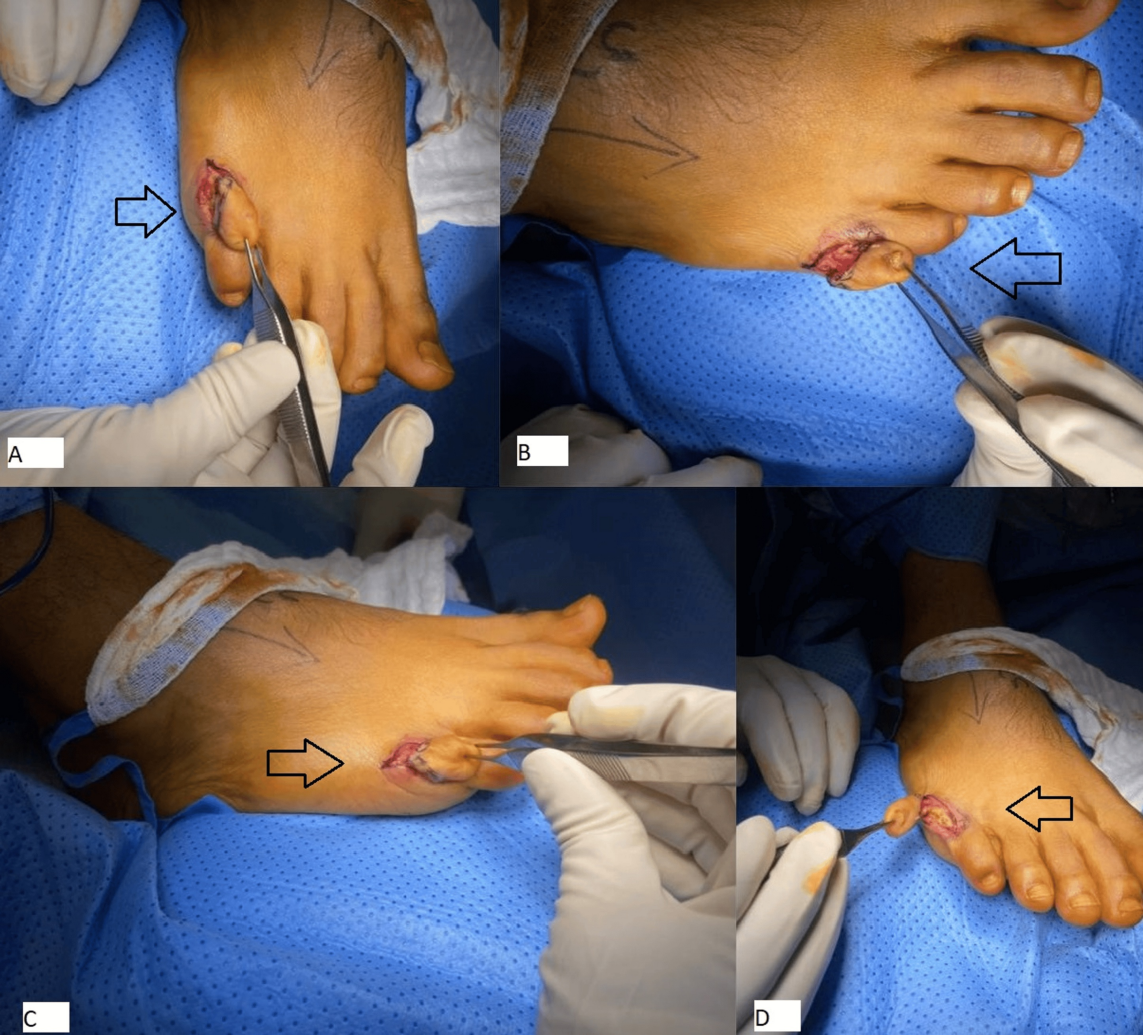
Right foot intraoperative pictures. (A-D) Intraoperative pictures highlighting the skin incision and various stages in the dissection.

A racquet-shaped skin incision was made around the extra digit. The incision was extended through the subcutaneous tissue, and the surrounding soft tissues were excised along with the extra digit. A small associated bony fragment was also removed. After releasing the tourniquet, hemostasis was achieved with bipolar cautery. The wound was irrigated with saline and closed in layers, using 2-0 nylon for the skin. A pressure dressing was applied (Figure 2).

The patient recovered well postoperatively and was discharged the same day with pain medications and instructions for wound care. He was seen two weeks postoperatively for suture removal, at which point the wound was healing well with no signs of infection.

Left Foot Surgery (September 2024)

The second surgery, for the excision of the left extra digit, was performed in September 2024. Similar to the first surgery, the patient was cleared for surgery in the PAC. In the operating room, general anesthesia was administered, and prophylactic cefazolin was given. A tourniquet was applied to the proximal thigh, and the foot and ankle were prepared and draped in a sterile fashion.

**Figure 3:**
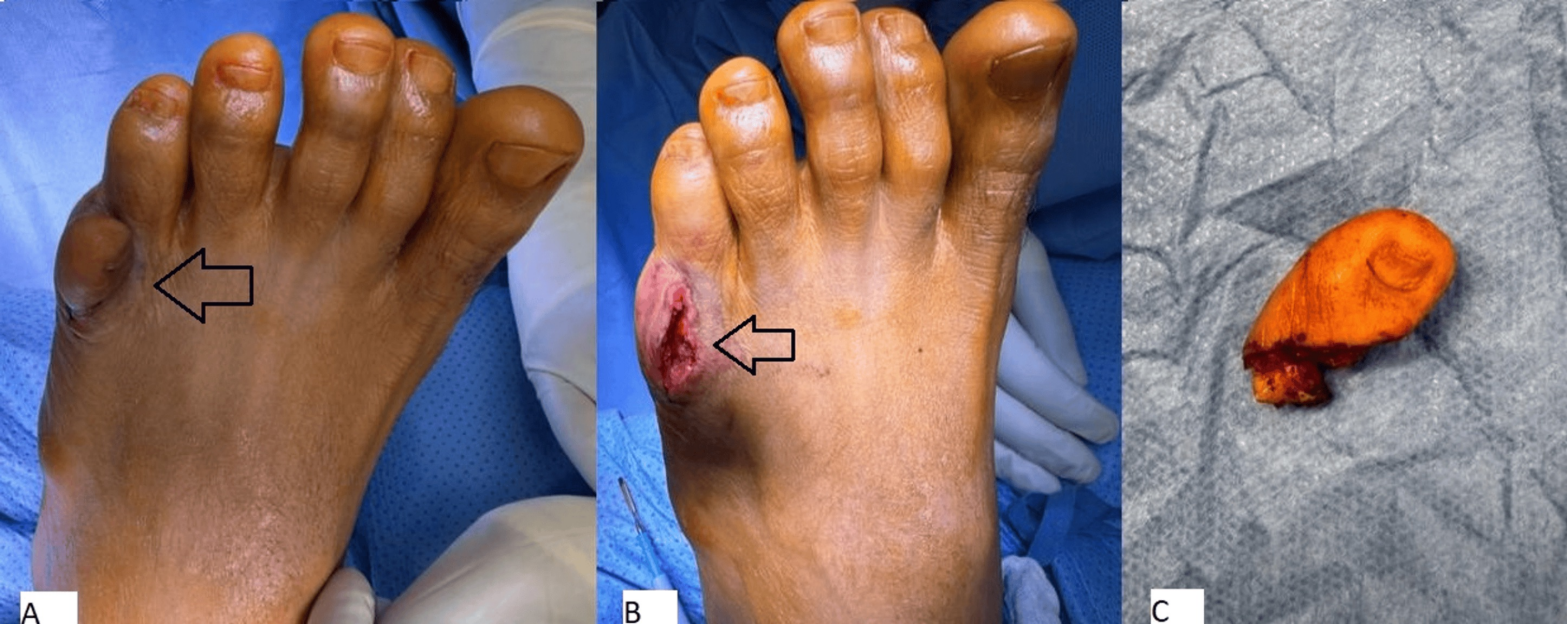
Left foot intraoperative pictures. (A) Before the incision. (B) After the removal. (C) The removed toe.

A racquet-shaped incision was made around the extra digit, extending through the subcutaneous tissues. The associated soft tissues and a small bony fragment were excised. Hemostasis was achieved with bipolar cautery, and the wound was irrigated thoroughly with saline. The wound was closed in layers, and the skin was sutured with 2-0 nylon. A pressure dressing was applied, and the patient was transferred to the recovery room in stable condition (Figure 3).

The patient was discharged on the same day with postoperative care instructions. He returned three weeks after surgery for suture removal, and the wound was healing appropriately without complications.

Postoperative course

**Figure 4:**
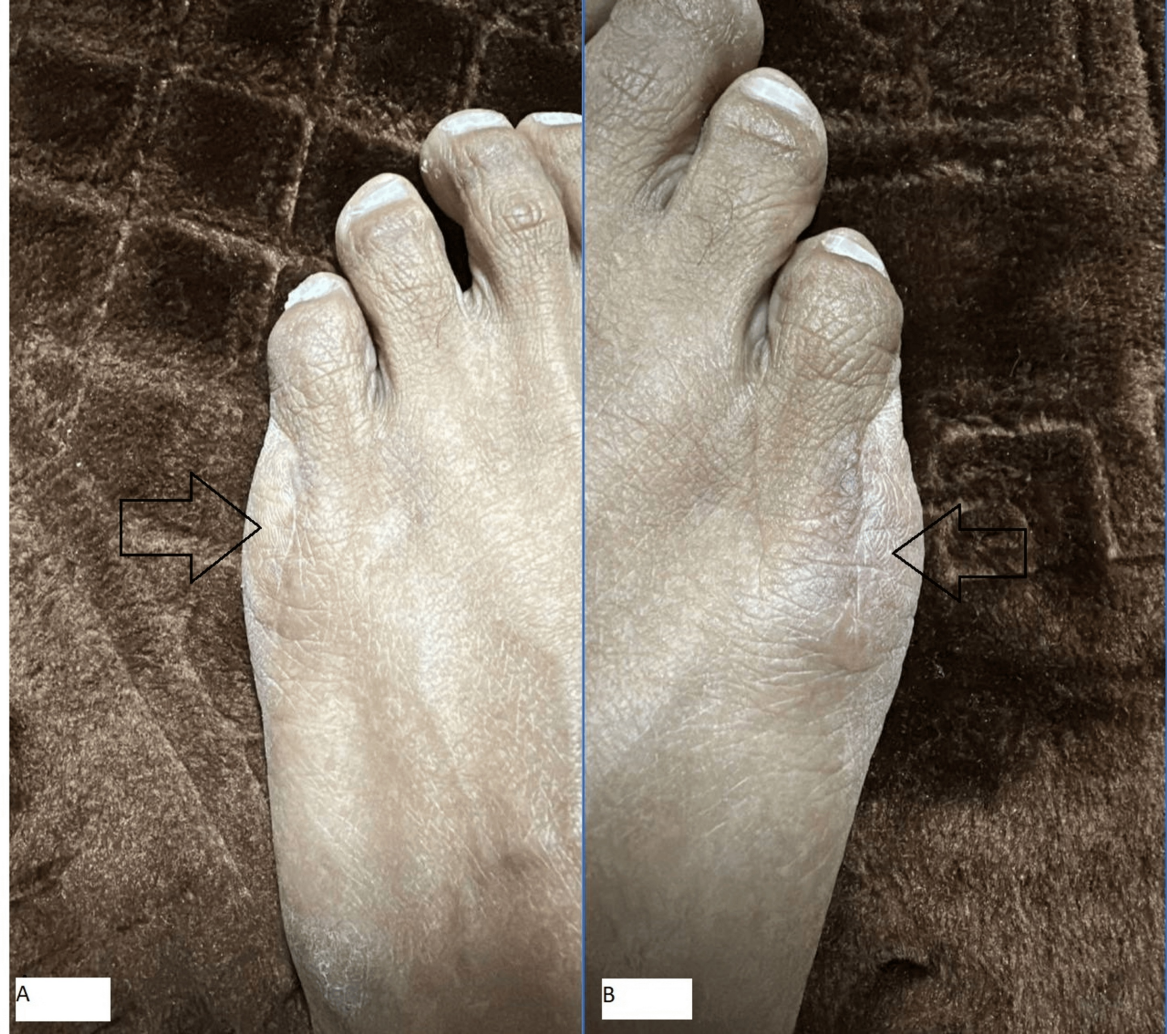
Surgical wounds one year after the operations. (A) Left foot. (B) Right foot.

The patient was seen for suture removal after each surgery. In both cases, the surgical sites were healing well, with no signs of infection or wound complications. The patient reported minimal postoperative discomfort and resumed ambulation without difficulty. The patient’s final follow-up was one year after the second surgery, where the patient showed full recovery with no residual discomfort and a cosmetically satisfactory result (Figure 4).

## Discussion

Polydactyly is a relatively common anomaly of the foot, although its symptoms can vary depending on the location and association with syndromes [4]. Postaxial polydactyly is less common than preaxial polydactyly, with bilateral involvement being particularly rare [3]. The presented case is an unusual presentation of bilateral type A postaxial polydactyly in an otherwise healthy patient.

The management of postaxial polydactyly depends on the morphology of the accessory digit, associated symptoms, and cosmetic considerations. Type B polydactyly, with no bony component, may be managed in infancy with simple ligation or surgical excision [4]. In contrast, type A polydactyly, where a bony element is present, requires formal surgical excision [4]. Our patient demonstrated osseous fragments within the accessory digits, confirming a type A classification, and reported shoe-wear difficulties and pain with prolonged ambulation, strong indications for surgical intervention in adulthood.

The surgical approach in this case employed a racquet-shaped incision, which facilitates complete excision of the digit, adequate soft tissue coverage, and a cosmetically favorable scar. Both procedures were uncomplicated, and the patient achieved excellent functional and cosmetic outcomes with no complications on follow-up. This supports previous reports, which showed that early excision, particularly when symptomatic, reduces long-term morbidity by preventing abnormal pressure points, callous formation, and gait disturbance [11].

The rarity of bilateral, isolated postaxial polydactyly of the feet makes this case valuable for the existing literature. It demonstrates that, even in adulthood, surgical excision provides excellent results with minimal morbidity.

## Conclusions

Bilateral postaxial polydactyly of the feet is an exceptionally rare congenital anomaly, particularly when it occurs in isolation and without syndromic associations. This case highlights the importance of recognizing such variations, as even long-standing, asymptomatic anomalies can become symptomatic in adulthood due to footwear pressure or altered biomechanics. Surgical excision can offer excellent functional and cosmetic outcomes with minimal morbidity. Our patient’s recovery highlights that proper surgical technique can achieve satisfactory results even in adult presentations. By documenting this case, we aim to contribute to the limited pool of literature on bilateral postaxial foot polydactyly and emphasize that individualized assessment and timely intervention remain key to optimizing patient outcomes.
